# The cost of being qualified: Current barriers faced by graduate students in forensic anthropology

**DOI:** 10.1002/ajpa.25005

**Published:** 2024-07-20

**Authors:** Thomas A. Delgado, Randi M. Depp, Raphaela M. Meloro, Katherine M. Lane

**Affiliations:** ^1^ Department of Geology and Geophysics University of Utah Salt Lake City Utah USA; ^2^ Department of Biology The University of Akron Akron Ohio USA; ^3^ Department of Anthropology University of Florida Gainesville Florida USA; ^4^ Department of Anthropology University of Central Florida Orlando Florida USA

**Keywords:** higher education, forensic anthropology, accessibility, diversity, equity, inclusion

## Abstract

When considering the best ethical practices in forensic anthropology, one must consider how accessible the field is to new students that are responsible for driving the future of research, pedagogy, and the field as a whole. While there is no denying that there are multiple barriers to accessing academia (e.g., racism, sexism, xenophobia, etc.) the cost of a graduate education is a key factor that affects the diversity of people that are able to enter the field. Here, the cost of 24 universities prominent in the education of forensic anthropologists are considered in tandem with the opportunities for funding offered by these institutions and average costs of living for the respective surrounding areas. Demographic data for the universities at the graduate and undergraduate level was additionally compared with the demographics of the cities surrounding the universities. Funding, excluding loans, was shown to be greatly below cost of living in university cities, and often did not match the costs of attendance estimated by institutions. Including the cost of living, the average graduate degree costs over $60,000 per year while the average stipend for graduate students is below $14,000 necessitating the need for loans or out‐of‐institute support. White individuals were overrepresented in graduate enrollment when compared with surrounding area demographics, even when university demographics were similar to those of the surrounding area. Overall, findings highlight the inaccessibility of pursuing higher education for minority groups and demonstrate the need for institutions to develop funding programs to promote diversity in higher education.

## Graphical abstract

Proportion of cost of living covered by graduate.

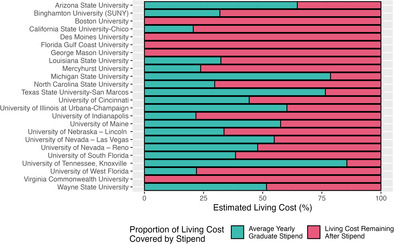

## 1 INTRODUCTION

The development of best ethical practices within forensic anthropology is an ongoing discussion traditionally encompassing topics such as research, casework, methodologies, sociopolitical concerns, and skeletal collections. However, the accessibility of higher education and the crucial role of graduate students in shaping the field's future are often overlooked. There are many sociopolitical barriers that individuals may face (e.g., racism, sexism, xenophobia, etc.) while attempting to access forensic anthropology graduate programs (Tallman et al., 2022). Among these barriers is the cost of attendance (COA), which includes tuition and fees, and the cost of living (COL). This financial burden likely plays a significant role in determining the diversity of graduate students and professionals currently active in forensic anthropology (Tallman et al., 2022). In recent years, this barrier of cost has grown substantially, and forensic anthropology programs, along with their affiliated faculty, should be keenly aware of the impact financial burden has on graduate students and how those burdens impact the field as a whole.

Recent events have exacerbated the financial burdens impacting graduate students in the United States. This includes the SARS‐CoV‐2 (COVID‐19) pandemic, which drastically disrupted how academia and the entire world operates. While the current employment market is rebounding (Cox, 2023), many young adults faced a sudden reduction or loss of employment options, including flexible, part‐time positions (e.g., campus jobs, food service, and retail industries) during the COVID‐19 pandemic (Brown, 2023; Browning et al., 2021; Carnevale, 2023; Keeter, 2020). During this time, many university students experienced heightened distress over personal finances (Browning et al., 2021; Keeter, 2020). These financial concerns persist since 2020 and are compounded by skyrocketing inflation, further wage stagnation, and an increase in interest rates and student loan debt burdens. This places graduate students in a perilous state of financial insecurity and reduced mental well‐being. In academia, these burdens disproportionately affect underrepresented minority (URM) students, ultimately increasing the inequality of access and retention at all levels.

From January 2020 to October of 2023 inflation increased 19% (U.S. Bureau of Labor Statistics, 2023a) and hourly wages have not kept pace with inflation. This confluence of rapid inflation with stagnant wages has left many households with insufficient funds for daily necessities (Brown, 2023; Egan, 2023). For graduate students, these realities are further exacerbated by limited employment opportunities and a rising COL. Recent faculty and graduate student labor strikes across the country highlight these swiftly growing differences which have resulted in the further disparity between academic salaries and the COL (El‐Bawab, 2022; Hall & Schermele, 2023).

In the United States, graduate students are frequently employed as graduate assistants (GAs; including research and teaching assistants). Typically, these positions are half‐time appointments requiring 20 h of work per week, dedicated to either teaching or conducting research. In return, GAs receive a monthly stipend, along with the potential for a tuition waiver and health insurance coverage. However, for the fiscal year 2021–2022, the mean GA stipend was just over $19,000, with little to no increase over the previous year (NEA, 2023). This wage stagnation leaves graduate students unable to cover basic needs like food, housing, and health‐related costs (Blake, 2023; El‐Bawab, 2022; McKibben et al., 2023; Mowreader, 2023; Sternstein & Pruett, 2023). More than 12% of graduate students experience low food security, and over 8% experience marginal food security, with significantly higher rates among Black graduate students, women, those who identify as LGBTQIA+, those with disabilities, and parents (McKibben et al., 2023). Additionally, 4.6% of graduate students reported being unhoused in the past month (McKibben et al., 2023). This trend is likely driven by the soaring rent‐to‐income ratio, with the average rent now requiring around 30% of an individual's income (Chen & Le, 2023), further exacerbating the housing needs of graduate students with wages below the COL.

The United States also has some of the highest costs for higher education worldwide (Cooper, 2019). This is coupled with a decades‐long shift in higher education cost‐sharing, transitioning the burden from governments and taxpayers to students and parents (American Academy of Arts & Sciences, 2015), and a nearly 5% increase in tuition rates in recent years (Dickler, 2022; U.S. Bureau of Labor Statistics, 2023b). Students now often rely on student loans to cover both their tuition and to subsidize their COL (Dickler, 2022; Palmer, 2023; Yaskowski, 2020). Simultaneously with the increase in student loan reliance, interest rates for these loans have hit a 10‐year high with rates for Direct Unsubsidized Loans and Direct PLUS Loans for graduate or professional students during the 2023–2024 academic year set at 7.05% and 8.05%, respectively (Minsky, 2023; U.S. Department of Education, 2023). Higher interest rates result in the addition of thousands, sometimes tens of thousands, of dollars over the lifetime of these loans (Minsky, 2023), negatively impacting those who must borrow more. While borrowing has increased among graduate and professional students in the recent decades, master's students have especially increased their borrowing (Pyne & Grodsky, 2020) potentially due to funding disparities between master's and doctoral students.

With students facing higher debt burdens than ever before, many are beginning to question their job prospects, the ability to repay their loans, and the benefit of higher education (Hiler et al., 2021; Macchiarola & Abraham, 2010). Graduate students intent on remaining in academia are currently facing historically low faculty salaries due to inflation, a trend that disproportionately affects women who continue to experience lower pay within the academic system (NEA, 2023). With financial impacts accumulating throughout undergraduate and graduate degrees, it is imperative that academic programs consider which students, and how many, are being priced out of their educational goals and potential careers. While living costs and tuition rise every year, graduate funding, wages, and support remain stagnant at best. At the same time, needs to standardize and professionalize forensic anthropology are requiring more education and certification (Passalacqua & Pilloud, 2020), ultimately increasing the cost of receiving essential training while wages for professionals remain undervalued (Passalacqua et al., 2020). This raises the question of whether it is ethical for departments to accept students for whom they cannot provide adequate financial support. Here, we explore the real cost of achieving a graduate education from universities closely tied to the discipline and highlight the unmet needs of graduate students pursuing forensic anthropology.

## 2 MATERIALS AND METHODS

An accreditation that many practicing forensic anthropologists seek or maintain is a diplomacy with the American Board of Forensic Anthropologists (D‐ABFA). Due to the perceived prestige of this accreditation, institutions that employ diplomates are considered to provide the highest quality of forensic anthropological training. This training often goes beyond the classroom and can include the ability to participate in forensic casework, a major factor that attracts students to those particular institutions. In viewing universities that employ diplomates of the ABFA as the leading institutions for forensic anthropological training, universities that employ at least one D‐ABFA on a full‐time (i.e., non‐Adjunct) basis that participates in the training of graduate students were considered for this analysis. This was further constrained to universities offering a graduate level (i.e., master's and/or doctoral) degree which is often required to participate in any form of medico‐legal death investigation as a forensic anthropologist.

From the ABFA's registry of diplomates, 24 universities offered graduate degrees in either anthropology (*n* = 17) or a closely related field (e.g., Biology; *n* = 7). Information available from the ABFA was then compared with institutional degree offerings via each universities' websites. In a few cases, additional graduate degrees were offered by the department that were not included in the ABFA registry and these were included in our dataset. Of the 24 universities in the dataset, 10 offered a terminal master's degree in anthropology or a related field, three only offered a doctoral degree, and 11 offered both master's and doctoral degrees (Table 1).

**TABLE 1:** Academic programs with at least one D‐ABFA as faculty and the program type, degree(s) offered, estimated annual cost, average annual stipend for GAs, and available healthcare subsidies.

University	Type	Program	Degree(s) offered	Estimated yearly cost^†^	Average annual stipend	Healthcare subsidy
Arizona State University	Public	Anthropology	PhD	$64,778	$24,586	Full
Binghamton University (SUNY)	Public	Anthropology	MA; PhD	$61,568	$10,779	None
Boston University	Private	Forensic Anthropology	MS	$1,08,917	$0	Partial
California State University‐Chico	Public	Anthropology	MA	$56,898	$7290	*
Des Moines University	Private	Anatomy	MS	$43,783	$0	Unspecified
Florida Gulf Coast University	Public	Forensic Studies	MS	$59,651	–	Unspecified
George Mason University	Public	Forensic Science	MS	$62,162	–	Unspecified
Louisiana State University	Public	Anthropology	MA; PhD	$62,874	$10,800	Partial
Mercyhurst University	Private	Anthropology	MA	$54,566	$7500	Unspecified
Michigan State University	Public	Anthropology	PhD	$62,702	$25,800	Full
North Carolina State University	Public	Biology	PhD	$70,102	$11,308	Full
Texas State University—San Marcos	Public	Anthropology	MA; PhD	$37,504	$16,173	Partial
University of Cincinnati	Public	Anthropology	MA	$58,554	$14,134	Full
University of Illinois at Urbana—Champaign	Public	Anthropology	MA; PhD	$61,399	$22,080	Partial
University of Indianapolis	Public	Biology	MS	$44,777	$7200	*
University of Maine	Public	Anthropology	MA; PhD	$63,093	$18,500	Partial
University of Nebraska—Lincoln	Public	Anthropology	MA	$50,289	$10,930	Partial
University of Nevada—Las Vegas	Public	Anthropology	MA; PhD	$59,127	$19,250	Full
University of Nevada—Reno	Public	Anthropology	MA; PhD	$49,478	$17,325	Full
University of South Florida	Public	Anthropology	MA; PhD	$53,076	$14,331	Partial
University of Tennessee, Knoxville	Public	Anthropology	MA; PhD	$62,029	$25,868	Full
University of West Florida	Public	Anthropology	MA	$51,410	$7200	*
Virginia Commonwealth University	Public	Forensic Science; Integrative Life Science	MS; PhD	$65,235	–	None
Wayne State University	Public	Anthropology	MA; PhD	$64,761	$18,909	Partial

^**†**^ Estimate includes out‐of‐state tuition and cost of living.

* Indicates that no student health insurance plan is offered.

For each program, data related to the COA, an adjusted COL, departmental funding and stipends, and demographic information was collected. COA information was collected directly from each universities' websites and was often in the form of annual or semester cost. When COA was instead determined by the number of credit hours a student was enrolled in (e.g., the University of Indianapolis), it was estimated assuming a nine credit‐hour course load per semester. COA also included estimated fees as provided by each university. Data related to the COL in the city where a university is located was collected utilizing the Massachusetts Institute of Technology Living Wage Calculator (Glasmeier, 2023) and assumed the COL for one adult with no children.

Data related to funding was limited to specific departmental funding opportunities based on avenues for graduate employment (e.g., GAs) and did not include external or internal funding via fellowships, grants, or other scholarships as they do not represent stable positions within a graduate program. While graduate students may acquire these forms of funding, students cannot rely on them when deciding to pursue higher education as they are typically dispersed after matriculation into a graduate program. Analyses of these funding opportunities are further complicated by the limited availability of data associated with their distribution, availability, and stipend allotment. Data concerning graduate employment were often publicly available through university websites or specific departmental graduate student handbooks. In cases where specific employment details dictating graduate student salary were not available, funding was estimated on the university's minimum stipend, assuming a 9‐month appointment with a 50% time commitment which aligns with the majority of university employment contracts. Frequently, stipend amount varied based on degree level (i.e., master's students receiving a lower stipend than doctoral students) or position level (i.e., teaching assistants receiving a higher stipend than research assistants) and in those instances the stipend was averaged.

Some universities offer Student Health Insurance Plans (SHIP) for their graduate students. Therefore, data were also collected regarding the cost of SHIP to graduate students funded through GA positions, as some universities offer subsidized or free healthcare to GAs employed with the university. This information was collected directly from university websites, their partner healthcare websites, and/or student handbooks. Recorded data included whether a SHIP was offered, the annual cost of SHIP, and the percentage subsidized for graduate student employees, if available.

Demographic information was collected relating to each university as a whole as well as the surrounding area. Whole university demographics were used as opposed to specific department demographics due to the inherent pervasiveness of university life while completing a degree thus providing a more holistic insight into daily interactions with the broader university system. Demographic data was collected using government data curated by the Data USA (Hidalgo, 2023) website. Racial categories provided by Data USA were slightly modified for the purpose of this research with their “Hispanic or Latino” category being renamed “Latine,” their “Non‐Resident Alien” category being renamed “Non‐U.S. Resident,” and their “American Indian or Alaskan Native” and “Native Hawaiian or Other Pacific Islanders” categories being grouped together and reclassified as “Indigenous (Including Pacific Islanders)” due to their low overall presence in the data.

Data were analyzed in the R programming language with graphics also being generated within R. Universities were compared to each other with respect to their COA and living, funding amount and availability, and demographics to provide realistic insights into the costs of attending a graduate program with a focus in forensic anthropology and the demographic representations that might be encountered while ingrained in a university system. Notably, this analysis does not include demographic data or discussion related to sex or gender that could also be considered barriers in achieving or retaining a graduate education. This highlights that more work needs to be done to address the intersections of identity, cost (both financial and psychological), and graduate training.

## 3 RESULTS

### 3.1 Cost of attendance, healthcare, and funding

Of the 24 universities surveyed, the average cost of annual attendance for out‐of‐state students before COL was $25,306 (*σ* = $9916). The average COL in a city with one of these universities was $34,989 (*σ* = $5093). When considered in tandem, the average estimated annual cost of attending a graduate institution was $60,117 with some variation present (*σ* = $13,568, *M* = $60,525). In‐state students received a considerable discount with in‐state tuition averaging $14,447 (*σ* = $10,868). When combined with COL, annual attendance for in‐state students averages $49,257 with some variation (*σ* = $13,976, *M* = $47,160).

Twenty‐one of the universities offered some form of a SHIP with 14 of these offering either a full or partial subsidy of the plan as a benefit of funding. The three universities that did not offer a SHIP were: CSU‐Chico, the University of Indianapolis, and the University of West Florida. The average cost of a SHIP was $3833 (*σ* = $1643) per year or approximately $320 per month with the average subsidy covering 67% of this cost. When considering the average subsidy, the cost of a SHIP was reduced to approximately $1265 per year ($106/month). Two universities (Virginia Commonwealth and SUNY‐Binghamton) offered no subsidy and four universities (George Mason, Florida Gulf Coast, Des Moines, and Mercyhurst) did not have information relating to a subsidy publicly available. There was a wide range of subsidization provided for the SHIPs offered, with seven universities offering 100% subsidies, five offering between 50% and 99%, and four offering below 50%. The University of Illinois Urbana‐Champaign provided a partial subsidy but the exact proportion was unspecified.

Funding information was not available from Florida Gulf Coast University, George Mason University, or Virginia Commonwealth University and those universities were not considered in these analyses. Two universities (Boston University and Des Moines University) offered no graduate funding and were included in these analyses. Of the 21 universities where funding information was available, the average graduate stipend was $13,808 with some variation (*σ* = $7594, *M* = $14,134). In assuming a 9‐month appointment at a 50% time‐commitment (20 h per week), the average hourly wage for graduate students was $19.18. In removing those universities that offer no funding, the average graduate stipend rose to $15,261 (*σ* = $6377, *M* = $14,331) resulting in an hourly wage equivalent to $21.20.

Importantly, this analysis does not include additional benefits such as tuition waivers as availability of these benefits were inconsistent between universities. Further, while tuition waivers do impact overall student debt burden, they and similar benefits do not directly impact a student's COL. This type of funding is not distributed directly to the student and cannot be used for any purpose other than for coursework. Of the 24 universities, three did not offer any form of tuition waiver (Boston University, Des Moines University, and Virginia Commonwealth University) Additionally, some tuition waivers (e.g., CSU‐Chico) only cover the difference between in‐ and out‐of‐state tuition and are not offered for the entirety of enrollment.

**FIGURE 1:**
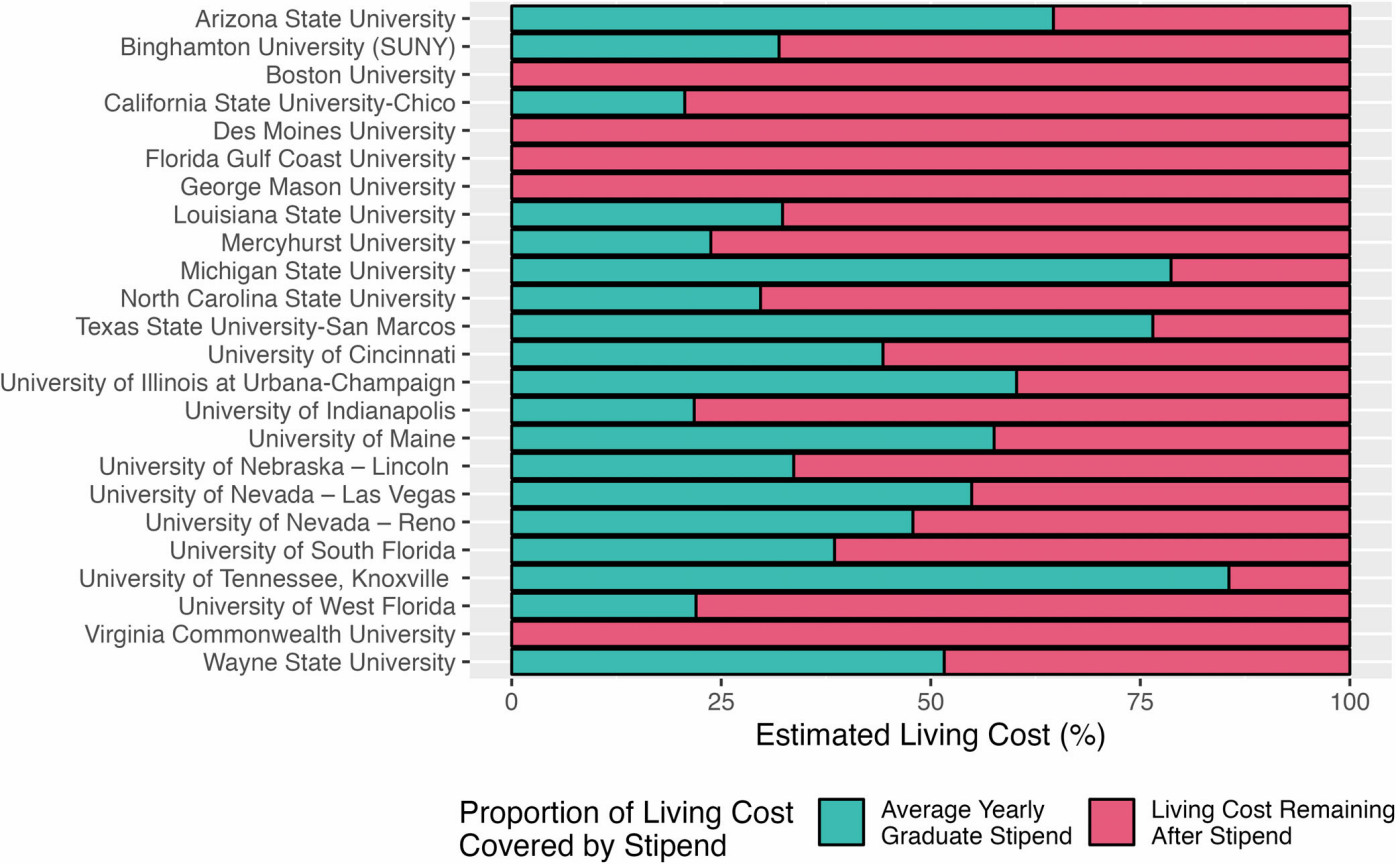
Proportion of cost‐of‐living covered by each University's yearly graduate stipend. Funding information was not available for Virginia Commonwealth University, George Mason University, or Florida Gulf Coast University. Funding information for Boston University and Des Moines University was available with no stipend being offered.

To account for the majority of universities that did offer some form of tuition waiver—both full or partial—the stipends for each university were compared with the COL in that university's city without considering the COA (Figure 1). Of the twenty‐one universities where funding information was available, eight of them provided a stipend that covered over 50% of their respective COL and, of those, three of them provided a stipend that covered over 75% of the COL. On average, including those institutions that provide no funding, graduate stipends cover just over 41% of a student's expected COL. This rose to just over 46% when universities that offered no funding were removed.

### 3.2 University and surrounding area demographics

When considering the demographic composition of each university and the surrounding area, those that identified as White were the most represented racial category in both universities and their surrounding areas. On average, those identifying as White composed 57% of the student population at these universities and composed 58% of the surrounding area population. Simultaneously, those identifying as Black composed 18% of the surrounding area population while contributing only 7.4% to the university student population. Those who identified as Latine displayed more even representation composing 11% of the surrounding area population and 14% of the university student population. Indigenous peoples were the least represented in both populations averaging approximately 0.4% of each population.

Notably, several universities had higher than average representation of minority groups resulting in some skew of the data specifically for those identifying as Latine. When considering the median of these populations, those identifying as Latine composed 9.3% of university populations and those identifying as Black composed 6.7% of university populations. In comparison, the median contribution by those identifying as White was 58% of university populations. For the surrounding areas, those identifying as Black composed 16% of the population, those identifying as Latine composed 7.6%, and those identifying as White composed 55%.

**FIGURE 2:**
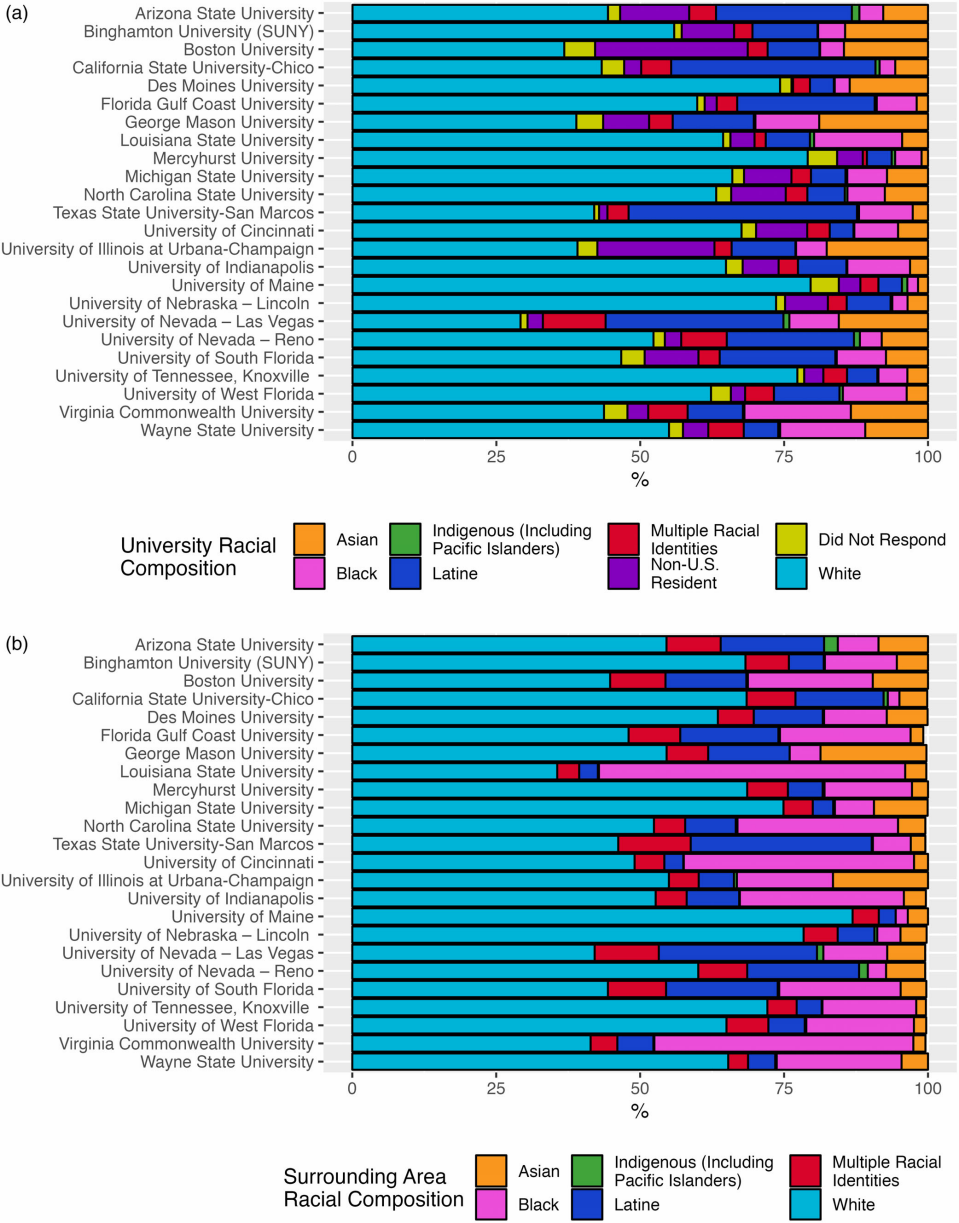
Demographic composition of each university (a) and its surrounding area (b).

Within the University system, approximately 7% of students—on average—were classified as Non‐U.S. Residents and specific demographic information for those students were unavailable. There was considerable skew in the amount of Non‐U.S. Resident students present between universities with two universities (Boston University and University of Illinois) maintaining an enrollment of 20% or greater in this category. In addition, approximately 3%—on average—of students did not self‐report their racial identity. Figure 2a provides a comparison of each specific university's demographic composition and Figure 2b provides a comparison of each university's surrounding area demographic composition.

## 4 DISCUSSION

### 4.1 The burden of cost

Financial security remains one of the primary concerns for graduate students worldwide with 85% of students worried about paying monthly rent or buying food and 45% of students considering quitting their program of study (Woolston, 2020). Women and those from lower socioeconomic status (SES) are particularly susceptible to facing extreme financial insecurity (Browning et al., 2021; Hiler et al., 2021; Walsh et al., 2021). Our analysis serves to demonstrate this widening gap between graduate student funding in forensic anthropology programs and the rapidly increasing COL. The average yearly graduate stipend for the 21 institutions available for analysis is more than $5000 below the national average graduate student stipend in the United States (NEA, 2023). Three universities—Michigan State University, Texas State University San Marcos, and University of Tennessee, Knoxville—provide stipends that cover more than 75% of the COL for those regions. Of the remaining 18 universities, only five pay graduate students enough to cover more than 50% of the region's COL. This puts forensic anthropology students at a far greater financial disadvantage when pursuing their career and likely limits which students can afford such a path. Academic training to be a forensic anthropologist can take 6–10 years (American Board of Forensic Anthropologists, 2023) resulting in up to a decade of cumulative underpayment, potential medical insecurity, missed career earnings and their associated retirement savings, and a growing student loan burden.

While it is evident that a majority of programs do not provide adequate funding to cover the COL, there are some explanations for the lower average graduate pay when compared with the national average. In our analysis, stipends were averaged across both master's and doctoral programs, with the master's stipends tending to be noticeably less than doctoral stipends; however, this alone is unable to fully account for this difference as the NEA data also averages across graduate program types. The NEA (2023) reports that graduate wages were significantly higher for STEM fields with the highest earners belonging to engineering and biology programs. This creates some disparity for those seeking degrees in programs with an emphasis in forensic anthropology as, while very STEM oriented, it is most frequently housed within the social sciences. For universities with lower overall budgets, the allotments provided to traditional STEM fields can be expected to be higher than allotments for the social sciences where forensic anthropologists are likely to be situated.

Our analysis focused on 24 institutions that range from R1 research universities to those with no research ranking. Due to variation in research rankings, the financial resources available to each institution will also vary; however, this should not be considered an acceptable reason to limit funding as universities frequently rely on the labor (and tuition) of graduate students. Of the three universities that provide a stipend above 75% of the COL, two are R1 institutions (University of Tennessee, Knoxville and Michigan State University). Simultaneously, of the remaining 21 universities, 14 are considered R1 institutions with 10 of these universities not providing a stipend that covers 50% or more of the COL and three providing no funding. This underscores the reality that even within R1 institutions with the highest expected research budgets, graduate students within forensic anthropology are unable to meet the rising COL.

On average, graduate students, regardless of discipline, carried more than $76,000 of total student loan debt in 2016, which amounts to more than $98,000 when adjusted for inflation (Hanson, 2023; U.S. Bureau of Labor Statistics, 2023a). Only 14.3% of this debt was incurred during the completion of undergraduate degrees (Hanson, 2023) illustrating the immensity of the financial burden required to pursue graduate studies. Master's students are particularly susceptible to financial strain with a 113% increase in borrowing since 2000 (Hanson, 2023; Pyne & Grodsky, 2020). For Master of Arts or Master of Science degree holders, the debt burden, as of 2016, was $92,300 and $79,000, respectively. This difference may be reflective of funding disparities among disciplines. For PhD graduates, the average debt climbs to $118,200 (Hanson, 2023), but as tuition, inflation, and student loan interest rates increase (Dickler, 2022; Hall & Schermele, 2023; Minsky, 2023), these numbers are likely to continue to increase.

Burgeoning interest rates are of particular concern for graduate students as graduate federal student loans have not been eligible for subsidization since 2012. This ensures that borrowers continue to accumulate interest even while enrolled in coursework (U.S. Department of Education, 2023). In order for students to manage their loan debt and prevent ballooning—where compounding interest rapidly increases the loan balance—it would be necessary for them to make regular payments on their student loans that would encapsulate the entirety of the interest and a small portion of the principal balance. For the majority of students this is an infeasible task. Our data demonstrates that graduate students, even in the highest paid funding positions, struggle to cover the COL, let alone a steadily increasing loan balance. This raises questions of how graduate students are making ends meet in light of the vast inadequacies in funding and limitations placed upon them within GA contracts.

Graduate assistantships are generally categorized as part‐time positions requiring 20 h of labor required per week in addition to fulfilling program requirements. One major caveat of GA contracts enforced by some universities is the limitation of the number of hours the GA is allowed to work for external employers, sometimes prohibiting this entirely. Even when students are able to work outside of their GA position, the additional hours necessary to complete program requirements results in graduate students working well over full‐time status. The terms of academic engagement as defined by 34 CFR § 600.2 set the guidelines for student participation at the federal level, giving states and institutions the freedom to adapt them as needed. Under 34 CFR § 600.2, a “credit hour” is expected to include 1 h of weekly instruction with an additional 2 h of external work put in by the student per week (Institutional Eligibility Under the Higher Education Act of 1965, 2023).

Thus, assuming a nine‐credit hour load per semester for graduate students, they are expected to put in a minimum of 27 additional hours of work per week to meet program requirements, placing them at 47 h of weekly “required” work per the GA contracts. These numbers still do not reflect the time necessary to complete data collection and analysis, preparation for candidacy exams and defenses, participation in forensic casework, or the large volume of reading and writing needed to complete a thesis or dissertation and publish research. When considering underfunded—both academically and socioeconomically—students' need to subsidize the COL with outside employment, there is easily the potential for individuals to be working upwards of 70 h each week simply to maintain their GA contract, a decent standing in their academic programs, and overcome inadequacies in funding via outside employment.

Unfortunately for many forensic anthropology students, these factors can be intensified through inconsistent funding per academic year, mixed availability for full or partial tuition waivers, no guaranteed funding for incoming students, and/or inadequate health coverage. It was apparent through the analyses of these data that there was rarely a funding package that addressed each of these concerns. These realities of inadequate and inconsistent funding, student overworking, and ever‐rising costs can create disparities within programs ultimately raising tensions between students and increasing incurred student debts overall. This added financial, physiological, and psychological stress has a profound effect on student mental health with graduate students experiencing depression and anxiety at higher rates than the greater population (Satinsky et al., 2021). This is especially true for URM students as they are disproportionately affected from the costs of student loans and financial stress with Black students bearing a majority of this burden (Belasco et al., 2014; Hahn & Tarver, 2023; Klink, 2022; Pyne & Grodsky, 2020; Yaskowski, 2020). In a field where students are, on average, paid below the national average and often require secondary employment to cover the COL, those from historically underrepresented groups may not be able to afford the training to become a forensic anthropologist.

### 4.2 Diversity and inclusion

While the cost of education can act as a significant barrier to all students considering entering the field of forensic anthropology, it has a disproportionate impact on URM students, most particularly those identifying as Black, Indigenous, and/or People of Color (BIPOC). This study found that the demographics of the universities examined rarely mirrored the demographics of their surrounding areas, with the universities generally having proportionally fewer BIPOC people than surrounding areas. Previous research has echoed these findings (Antón et al., 2018; Di Yim et al., 2022; Erhart & Spradley, 2022; Goliath et al., 2023; Tallman et al., 2022; Tegtmeyer Hawke & Hulse, 2023).

As mentioned in previous sections, several universities in the study had higher than average representation of minority groups resulting in some skew of the data specifically for those identifying as Latine. Three of the institutions surveyed—California State University, Chico; Texas State University, San Marcos; University of Nevada, Las Vegas—are federally recognized Hispanic‐serving institutions (HSIs; Erhart & Spradley, 2022), and the demographic breakdown of their programs is influenced by that of the surrounding community (National Center for Education Statistics, 2023). Additionally, these HSIs differ from other institutions by their increased focus on diversity and inclusion initiatives designed to create welcoming and safe spaces for BIPOC students. Unique opportunities offered at these HSIs, such as work with project Operation Identification, may draw more BIPOC students to apply to these programs.

As exemplified by these HSIs, the development of stronger diversity, equity, and inclusion (DEI) initiatives and better mentoring programs may help with drawing and retaining URM students in forensic anthropology. High‐quality mentorship by faculty and a supportive peer network has been demonstrated to improve retention outcomes for all graduate students across disciplines (Milosch & Tram, 2022). Recent research in biological anthropology, and forensic anthropology specifically, has called for the development of such programs and an enhanced focus on DEI initiatives to retain and support URM scholars (Antón et al., 2018; Di Yim et al., 2022; Goliath et al., 2023; Tallman et al., 2022; Tallman & Bird, 2022).

This is of particular importance to the field due to its history of racism and its affiliation with organizations that have historically treated minorities with discrimination and harassment (Antón et al., 2018; Tallman & Bird, 2022); however, these calls are not yet reflected across the forensic fields or prevalent at the professional level. In their study, Tallman and Bird (2022) surveyed American Academy of Forensic Sciences (AAFS) Anthropology section members, finding that 39% of respondents have witnessed discrimination at AAFS annual meetings. While the majority (63.4%) of survey respondents felt that there was a lack of diversity in forensic anthropology and that this lack of diversity was an issue (73.4%), a prior AAFS‐wide survey indicated that most (85.9%) AAFS members felt that the AAFS should not do more to promote minority participation in its activities (American Academy of Arts and Sciences, 2015; Tallman & Bird, 2022). Without strong multifield support in forensics, a truly supportive environment cannot be developed for URM scholars and professionals in forensic anthropology.

While improving DEI efforts will aid the retention of URM individuals in graduate programs, this alone cannot sufficiently address the lack of URM individuals in forensic anthropology without addressing the unequal financial burdens they face. All forms of financial aid impact student retention rates, but access to grant funding has been found to have the most significant impact on a student's choice to remain in a program (Gururaj et al., 2010; Milosch & Tram, 2022). While graduate assistantships are available at many institutions, their limited hours, funding, and inconsistency mean that they are not a reliable source of income for many individuals, including URM students. This is especially true for international students attending United States universities on F‐1 visas, which limit their employment to only 20 h per week (U.S. Immigration and Customs Enforcement, 2023) creating further disparity in overcoming their COL differences.

Loans may be an alternative and supplemental option for funding but, among both undergraduate and graduate borrowers, there are certain groups that carry a higher student loan burden including women, individuals from lower socioeconomic backgrounds, BIPOC, and those with dependents (Belasco et al., 2014; Hahn & Tarver, 2023; Klink, 2022; Pyne & Grodsky, 2020; Yaskowski, 2020). Additionally, BIPOC students receive far fewer private scholarships (Klink, 2022) and often lack generational wealth (Yaskowski, 2020) increasing the overall loan burden that these students must take on. In response to the lack of adequate funding and financial security, more students have begun to “underborrow” wherein they forgo their basic needs, the completion of their academic program, or rely on personal credit either due to reaching their borrowing limits or seeking to avoid student loan debt altogether (Ferguson, 2003; Sternstein & Pruett, 2023).

Overall, the burdens of a graduate education in forensic anthropology, especially costs and access to funding, place low‐income, first generation, immigrant, BIPOC, and other marginalized students at a greater disadvantage than their privileged peers (Tallman et al., 2022). Yet, it is crucial to address these issues for the future of the field (Di Yim et al., 2022; Tallman et al., 2022). The underrepresentation of minority students and professionals in forensic anthropology negatively impacts the quality of research and practice in the field, hinders innovation, and reduces connection with the communities that we serve.

### 4.3 Additional barriers

The growing disparity between COL and graduate funding creates or contributes to additional barriers that place significant burdens on more vulnerable and already burdened groups. More than 20% of graduate students face food insecurity (McKibben et al., 2023). Food insecurity and malnourishment negatively impacts academic performance and leads to reduced health and increased depression and anxiety (Sternstein & Pruett, 2023; Wang et al., 2019). Undernourished students are also less likely to graduate from their academic program or attain higher degrees (Wang et al., 2019; Wolfson et al., 2022). These issues are more likely to impact first‐generation students, individuals with a disability, nontraditional students, international and BIPOC students, LGBTQIA+ students, parents, and those of lower SES (McKibben et al., 2023; Wang et al., 2019; Wolfson et al., 2022). With the reduced funding available within forensic anthropology, this means that these groups may be even less likely to attain a degree.

Additionally, our data demonstrates that health insurance is not consistently available or subsidized for graduate students in the field. Several universities have hard‐waiver health insurance policies that require that students carry health insurance, where they either are automatically enrolled in a SHIP or must provide proof of acceptable coverage by a specified date (Kim et al., 2021). However, the requirements for insurance often vary based on the university, degree level (i.e., undergraduate vs. graduate), and residence status (i.e., US resident vs. international student). Often GAs, as university employees, are automatically enrolled into a SHIP, unless students submit a hard‐waiver that demonstrates comparable coverage. Additionally, several universities in our dataset mandated international students to carry health insurance, either through a SHIP or comparable plan. High cost of healthcare in the United States prevents individuals, especially from low‐income and marginalized groups, from seeking or obtaining necessary services and medications (Lopes et al., 2024). With some student insurance plan premiums costing additional hundreds or thousands of dollars on top of tuition, fees, and COL, health insurance and healthcare may be a luxury that further restricts access to graduate education. This disproportionately disadvantages those with dependents, chronic illnesses, and disabilities.

Mental health among graduate students is often poor (Posselt, 2021; Satinsky et al., 2021) due to systemic factors inherent in most graduate programs and institutions of higher learning (Bekkouche et al., 2021). While this phenomenon is known, little is often done to correct it. Universities provide psychological and counseling services to students, often at no charge due to already paid student health fees, but many are not established for the level and duration of support graduate students would require. This if further impacted as need for these services steadily increases. Nearly 12% of the student population utilizes their campus' counseling services, compared to under 7% in 2007 (Lipson et al., 2019). As this ratio increases, university systems could struggle to maintain the level of care required causing long waitlists, impacts to academic performance, and the potential to miss treating students that are in crisis. If a student requires further off‐campus care and/or higher levels of care (e.g., psychiatric services), then they may not be able to afford mental health treatment and medications. Students facing these financial barriers may be priced out of training in forensic anthropology simply to maintain their mental well‐being.

Issues of financial insecurity and mental health do not end at the graduate level. Forensic anthropology is a small field with limited job growth potentially creating financial stress for students graduating and trying to enter the workforce (Passalacqua et al., 2021; Tallman et al., 2022). Concerns over relatively low salaries may further exacerbate financial worries when trying to secure a job (Passalacqua et al., 2020). Students hoping to have their loans forgiven postgraduation through the Public Service Loan Forgiveness program still have to maintain payments for 10 years while employed in the public sector. This form of public service employment, while meaningful, is traditionally underpaid compared with private employment likely resulting in an additional decade of financial struggle. Forensic anthropologists also regularly interact with agencies with histories of discrimination and harassment of minorities as part of their work in academic and applied sectors. The findings of Tallman and Bird's (2020) survey of the AAFS Anthropology section regarding high rates of witnessing or being subject to discrimination and lack of diversity indicate these may be significant stressors in the workforce. Together, this culture of exclusion and the lack of financial security in forensic anthropology can combine to harm the mental health of professionals in the field.

## 5 CONCLUSION

Our findings demonstrate a common problem in academia: graduate students are underfunded and overburdened. This is especially clear in forensic anthropology, where graduate students are paid well below the national average. Importantly, even in the higher paid STEM positions there are still issues in addressing diversity problems and the resolution of the “leaky pipeline.” Many of the efforts made by STEM fields mirror those used within forensic anthropology; however, forensic anthropology appears to fail in retaining and recognizing minority scholars despite its higher percentages of women and BIPOC scholars entering the field (Dutton et al., 2017; Erhart & Spradley, 2022; Wagstaff & LaPorte, 2018). There is a lack of representation of diverse backgrounds with white men occupying a disproportionate number of positions of power within the field (Tallman & Bird, 2022; Ward et al., 2019). Paths to increased retention and well‐being then hinge on supporting URM scholars who are prone to lacking personal wealth. Increased support would necessitate the adjustment of graduate student stipends to better cover the increasing COL, increasing the accessibility to food resources and healthcare, and the creation of environments free of harassment to allow graduate students to thrive. As the discipline of forensic anthropology pushes for increased standardization, certification, and adherence to ethical codes, the ethics of graduate student treatment should not be overlooked.

The reality of limited applied forensic anthropology and faculty positions might also require programs to consider the ethics of accepting more students than for whom they can provide funding packages. While there is a great deal of nuance when making these decisions, consistent underfunding raises concerns about the diversity of scholars and the potential influence of those who can supplement their income through generational and/or personal wealth on the pedagogical, theoretical, and research orientations of the next generation of scholars. Further, our study highlights a lack of accessibility to program‐specific data relating to graduate funding packages. Prospective students should have the chance to make an informed decision about their future by considering their interest in the field, career prospects, and the cost of education before committing to a program. Without transparency on the part of the university regarding these costs they cannot fully do so. While universities do offer breakdowns of costs for classes and supplies, the authors of this paper found that university websites were not transparent or consistent in their information regarding graduate student funding opportunities—including the number of assistantships offered and their associated stipends. Overall, this research underscores the importance of transparent and equitable funding to foster a rich and diverse scholarship that will better serve the field now and in the future.

## AUTHOR CONTRIBUTIONS

**Thomas A. Delgado:** Conceptualization (equal); data curation (lead); formal analysis (lead); investigation (equal); methodology (lead); project administration (lead); resources (equal); software (lead); validation (equal); visualization (lead); writing – original draft (equal); writing – review and editing (equal). **Randi M. Depp:** Conceptualization (equal); data curation (supporting); formal analysis (supporting); investigation (equal); methodology (supporting); resources (equal); visualization (supporting); writing – original draft (equal); writing – review and editing (equal). **Raphaela M. Meloro:** Conceptualization (equal); data curation (supporting); formal analysis (supporting); investigation (equal); methodology (supporting); project administration (supporting); validation (equal); writing – original draft (equal); writing – review and editing (equal). **Katherine M. Lane:** Conceptualization (equal); data curation (supporting); formal analysis (supporting); investigation (equal); validation (supporting); writing – original draft (equal); writing – review and editing (equal).

### OPEN RESEARCH BADGES

This article has earned an Open Data badge for making publicly available the digitally‐shareable data necessary to reproduce the reported results. The data is available at https://osf.io/qvgyz/YOI10.17605/OSF.IO/QVGYZ/.

## Data Availability

Data collected for this project has been made publicly and freely available for additional review and analysis under a Universal Creative Common License. Data is hosted through the Open Science Framework repository and can be accessed with the following link: https://osf.io/qvgyz/ or found with the following: DOI 10.17605/OSF.IO/QVGYZ.
